# Advancing care priorities for health and quality of life among older adults in the autism and/or intellectual disabilities communities: proceedings of an international Think Tank

**DOI:** 10.1186/s12919-025-00330-8

**Published:** 2025-06-16

**Authors:** David B. Nicholas, Fakhri Shafai, Stephen M. Edelson, Vanessa Bal, Hilary Nelson, Wenn Lawson, Mary Doherty, Hilde M. Geurts, William F. Sullivan, B. Blair Braden, Gregory L. Wallace, Maxine Share, Laura St. John, David G. Amaral, Agnes H. Whitaker, Lori Watters, Terri Robson, Joanne Gauthier, Sylvère Moulanier, Linda Perry, Sandy Stemp, Ellen Wilkinson, Nancy Jokinen, Margaret L. Bauman, Robert Hendren, Evdokia Anagnostou, Julian N. Trollor

**Affiliations:** 1 Faculty of Social Work, Central and Northern Alberta Region, University of Calgary, Edmonton, Canada; 2 AIDE Canada, Richmond, Canada; 3 Autism Research Institute, San Diego, USA; 4 Graduate School of Applied and Professional Psychology, Rutgers University, New Brunswick, USA; 5 Curtin Autism Research Group, Curtin University, Western Australia, Perth, Australia; 6 School of Medicine, University College Dublin, Dublin, Ireland; 7 Brain & Cognition Department of Psychology, Faculty of Social and Behavioural Sciences, University of Amsterdam, Amsterdam, the Netherlands; 8 Kennedy Institute of Ethics, Department of Family Medicine, School of Medicine, Georgetown University, Washington, DC USA; 9 College of Health Solutions, Arizona State University, Phoenix, USA; 10 Department of Speech, Language & Hearing Sciences, The George Washington University, Washington, DC USA; 11 Autistic Consultant and Writer, Ontario, Canada; 12 Faculty of Kinesiology, University of Calgary, Calgary, Canada; 13 Department of Psychiatry and Behavioral Sciences, The MIND Institute, University of California-Davis, Davis, USA; 14 Department of Psychiatry, Columbia University Irving Medical Center, New York, USA; 15 Alberta, Canada; 16 British Columbia, Canada; 17 Vela Canada, Langley, Canada; 18 Reena, Thornhill Canada; 19 Department of Psychology, Rutgers University, New Brunswick, USA; 20 School of Social Work, University of Northern British Columbia, Prince George, Canada; 21 School of Medicine, Boston University, Boston, USA; 22 Department of Psychiatry, University of California, San Francisco, San Francisco, USA; 23 Faculty of Medicine, Holland Bloorview Kids Rehabilitation Hospital, University of Toronto, Toronto, Canada; 24 National Centre of Excellence in Intellectual Disability Health, UNSW Medicine and Health, UNSW Sydney, Sydney, Australia

**Keywords:** Autism, Intellectual disabilities, Aging, Seniors, Older adults, Health, Quality of life, Gerontology

## Abstract

As the number of older autistic adults and adults with intellectual disabilities grows, expanding capacity to meet their needs is crucial. On November 23-25, 2023, a Think Tank on aging in autism and/or intellectual disabilities was convened, with national and international delegates within this field. The Think Tank consisted of presentations focusing on key issues as well as a series of panel presentations addressing first-person lived experience, family caregiving, service provision in the community, and physical and mental health-based care. Discussion reflected lived experiences and care needs in this population, with an ultimate aim of advancing healthcare and community support. Delegates, who represent perspectives as self-advocates, family caregivers, service providers, clinicians and researchers, ranked guidelines and areas of focus identified in the literature in terms of the most important priorities for the near-term development of capacity building resources.

With a globally aging population, gerontology and geriatrics have been the focus of substantial recent research that spans physiologic, neurologic, social, and community considerations [1, 2]. However, the needs of those with autism and/or intellectual disabilities have been under-addressed in this research, as well as in practice and program advancement. Processes identified as needed in moving this field forward include bringing individuals with lived experience, community partners and scholars together, identifying key issues or areas for capacity advancement, and creating priorities for moving forward. To that end, an international meeting entitled, “Autism and/or Intellectual Disabilities and Older Adults: A Think Tank Promoting Quality of Life and Health” was held on November 23–25, 2023 in Vancouver, Canada. Participants comprised inter-disciplinary and inter-sectoral stakeholders committed to knowledge development and capacity building in this field. This Think Tank was the second in-person meeting of some members of this international group. The earlier meeting, convened in 2017 and summarized by Edelson et al. [3], advocated for capacity building and planning related to aging in autism. Seeking to advance this work, this subsequent 2023 Think Tank was attended by adults in mid- and later-life with first-hand lived experience of autism and/or intellectual disability, family caregivers, community support providers, community care clinicians/support workers, healthcare providers, advocates, program planners/administrators, trainees and researchers. In total, 45 individuals attended the Think Tank in-person, and 12 individuals joined via online streaming. Delegates were from Canada, the United States, Australia, Ireland, and the Netherlands. Think Tank goals aimed to advance knowledge and explore capacity building strategies for improved health and quality of life among older autistic adults and/or adults with an intellectual disability. Specific meeting objectives were to (1) identify key areas to advance health and quality of life outcomes for older individuals in the autism and/or intellectual disabilities communities, and (2) determine stakeholder priorities to inform future knowledge translation initiatives and capacity building. Based on priorities of ‘nothing about us without us,’ the meeting was widely attended by individuals with lived and living experience. This included in-person presentations as well as pre-recorded streaming of individual descriptions of experiences and recommendations relative to specific topics being addressed in sessions.

The conference offered keynote addresses, presentations and panels addressing salient topics regarding the health and quality of life of older autistic individuals and/or adults with intellectual disabilities. Presenter perspectives included lived experience, and key priority areas such as considerations in caregiving, community services, clinical care and research. Speakers addressed critical issues, advances and challenges, and offered recommendations for moving forward. Results of a scoping review of the literature examining health and quality of life among older autistic individuals and/or individuals with intellectual disabilities [4] were shared. The scoping review provided insights and context for reflection and discussion during Think Tank deliberations. Below, we briefly present the keynote addresses, presentations, panels and priority-ranking activity results. The following sections summarize key themes discussed in the Think Tank.

## Keynote addresses

Two keynote addresses were offered by international scholars. Dr. Wenn Lawson of Curtin University in Australia focused on monotropism, object permanence and interoception in older autistic adults, and conveyed the need for improved support for this population. Dr. Hilde Geurts of the University of Amsterdam addressed health and well-being related to aging in autism. Abbreviated summaries of these keynotes are presented below.

### Keynote address 1: interoception and object permanence in aging autistic adults

Wenn Lawson, PhD, AFBPsS, MAPs: Autistic Consultant; Independent Researcher; and Adjunct Associate Professor, Curtin University, Australia.

Dr. Lawson introduced the concepts of monotropism, interoception and object permanence to outline key, yet under-addressed, considerations in our understanding of autism. He argued that these notions are critical to consider in supporting physical and mental health and quality of life among many autistic seniors. Dr. Lawson noted that social and physical environments are adapted for some more than for others, and in particular, are less adapted for autistic people. He proposed increasing attention to how autistic individuals perceive and experience shared environments (e.g., grocery stores, healthcare spaces, etc.). He emphasized the challenge and exhaustion experienced by autistic individuals who are continually having to attempt to interpret and navigate environments designed for neurotypical persons. Integrating the concepts of monotropism, interoception and object permanence, Dr. Lawson discussed implications for the health and well-being of older autistic adults.

Monotropism or interest theory, which frames attention as a finite resource, addresses attention allocation in neurodivergent individuals compared to neurotypical individuals [5, 6]. Monotropic minds are proposed to allocate the majority of their attention to a singular task, leaving minimal space for attention to other matters, such as recognizing or responding to environmental changes, people or sensory input. As described, monotropism requires time and effort to navigate the ‘attention tunnel’ and realign attention (often through individual interests) to new tasks [5]. Interoception is the sense that allows individuals to identify, interpret and distinguish internal physical or emotional feelings [7], whereas object permanence is the awareness that the ability to sense or not sense an object, person, environment or season at a given time, does not necessarily affect its continued existence [8, 9].

Dr. Lawson shared examples of how monotropic autistic individuals may process experiences differently regarding health and well-being; for example, leaving before seeing a doctor because the scheduled appointment time has passed, not being able to identify or describe where physical pain is located, or not noting the difference between being out-of-sight (but still existing) and being gone from existence. Although monotropic minds may recognize, process and communicate feelings of discomfort, pain, grief and loss, or the desire for human connection in different ways, it does not mean that these feelings do not exist. Rather, implications of monotropism include the need for adaptations to shared environments in order to better support health and quality of life.

### Keynote address 2: aging in autism

Hilde Geurts, PhD, Brain & Cognition Department of Psychology, Faculty of Social and Behavioural Sciences, University of Amsterdam, the Netherlands.

Reflecting on research on cognitive aging and heterogeneity across the autism spectrum, Dr. Geurts addressed the impact of aging on the health and well-being of older autistic adults. She outlined the available aging research in this field, and reflected on the limited number of investigations to date. Summarizing her research, Dr. Geurts noted that earlier trajectories are important in discerning future needs and support requisites. Currently, there is contradicting evidence on whether autistic individuals are at heightened risk for faster cognitive aging. Dr. Geurts shared research results that suggest that autistic individuals are not at heightened risk for faster cognitive aging [10, 11], but there are other datasets that indicate potential increased risk or earlier onset of cognitive decline [12, 13]. Dr. Geurts noted that some autistic adults reflect what is referred to as an older age profile; that is, they report feeling older than they are in actual age [14–16].

In relation to her research on older autistic adults without intellectual disabilities, Dr. Geurts noted the following. In general, older age yields greater risk for age-related conditions (e.g., cardiovascular disease, elevated cholesterol, high blood pressure, dementia) as well as for issues such as slower information processing and more problem-solving difficulties. While these outcomes may indeed be challenging, well-being outcomes tend to conversely suggest that autistic individuals generally become slightly happier, may feel more in control of their lives, and/or may have desired elements (e.g. less demands) in later years. Yet with older age, significant changes unfold in the social environment such as shifts in the role/presence of caregivers. Multiple factors have a bearing on the aging process including genetic factors and social factors such as gender, sex, income (and its impact on lifestyle) and stress. Also deemed important are physical activity, support networks and social networks [11]. More research is needed to ascertain how aging may affect the full spectrum of autistic people, and understand the experiences and needs of family members and caregivers.

## Presentations

Four presentations were offered by international scholars. as follows. (1) William Sullivan, PhD, of the Kennedy Institute of Ethics, Department of Family Medicine, School of Medicine, Georgetown University in the United States, outlined the process of primary care guideline development for improved clinical care for adults with intellectual and developmental disabilities. (2) Mary Doherty, MD, of the School of Medicine, University College Dublin in Ireland, addressed the development of a framework to guide clinicians in advancing healthcare for autistic adults. (3) David Amaral, PhD, of the MIND Institute, Department of Psychiatry and Behavioral Sciences, University of California, Davis in the United States, addressed the value of brain tissue research and the importance of brain tissue donation at the end of life to build knowledge and capacity toward improved health and quality of life for autistic individuals. (4) Fakhri Shafai, PhD, M.Ed., of AIDE Canada, reflected on key principles and guidelines for moving knowledge to action and impact. Each of these presentations are summarized below.

### Presentation 1: development of primary care guidelines in intellectual and developmental disabilities

Dr. William F. Sullivan, Joseph P. Kennedy Senior Chair in Bioethics, Kennedy Institute of Ethics; Professor, Department of Family Medicine, Georgetown University, Washington, DC, USA.

Formerly the clinical lead of the Developmental Disabilities Primary Care Program at Surrey Place in Toronto, Canada, Dr. Sullivan and colleagues developed Canadian consensus guidelines for the delivery of primary care to adults with intellectual and developmental disabilities [17]. He noted that, “beginning in 2011, with each update of the guidelines, we published tools to help implement recommendations.” Supporting this process, Dr. Sullivan recognized joint funding for this initiative from the then Ontario Ministry of Community and Social Services and the Ontario Ministry of Health, reflecting that such inter-ministerial and cross-jurisdictional collaboration seems important in recognizing and acting on cross-disciplinary responsibility for, and wide-ranging facets of, this work. Moreover, Dr. Sullivan noted that with the closure of institutional housing for people with intellectual disabilities in Canada, the complex health needs of this population required skill development among primary care providers in the community. To develop primary care guidelines, Dr. Sullivan and colleagues embarked on a review of the literature, along with bringing together international experts in the field [17]. Based on the literature and a deliberative process, the determination of, and consensus on, guidelines (briefly summarized within Appendix) were achieved. Importantly, Dr. Sullivan noted strong participation by people with intellectual and developmental disabilities and family members, which was deemed to be integral to the guideline development process. A crucial issue of inclusion, noted Dr. Sullivan, “is adapting and accommodating needs and supporting decision-making so that [people with intellectual and developmental disabilities] can be as involved as possible.”

### Presentation 2: development of a healthcare framework in autism

Dr. Mary Doherty, Clinical Associate Professor, School of Medicine, University College Dublin, Ireland.

Dr. Doherty is the founder of Autistic Doctors International which, “brings together autistic healthcare providers who are dedicated to improving healthcare for autistic people and improving work life for autistic healthcare providers” [18]. She and colleagues created the “Autistic SPACE” framework for addressing the healthcare needs of autistic people [19]. Noted as also applicable to other settings such as education, justice, residential care, and home settings, this work was a response to previous research that identified challenging healthcare processes and experiences as well as adverse outcomes for autistic people [20]. Barriers have been documented such as difficulty with communication, accessing appointments, using public transportation to get to healthcare facilities, and the sensory environment in healthcare settings. The team identified that communication difficulties for some have led to additional challenges such as diagnostic overshadowing and difficulty accessing healthcare, possibly leading to healthcare avoidance. Dr. Doherty noted, “It was very clear to us, as doctors, what we were analyzing in this data…. These were very serious medical conditions, potentially life-threatening conditions for which people were not able to access healthcare.” She further noted, “it seemed quite plausible that there would be an association between the barriers to healthcare and adverse outcomes which may well potentially lead to reduced life expectancy for autistic people, and things like autistic people not accessing screening, and therefore potentially presenting for healthcare much later in the natural history of an illness so potentially presenting with late-stage cancer, for example.”

Of further concern, Dr. Doherty noted limited autism-related training for most medical professionals. For non-specialists, autism can seem quite complex and nebulous, with care providers finding it difficult to know an autistic individual’s care needs. This team concluded that clinicians needed a framework that is easily discernable and memorable, yet comprehensive, which led to the creation of the ‘Autistic SPACE’ framework [19]. SPACE is an acronym that stands for: Sensory Needs, Predictability, Acceptance, Communication, and Empathy (briefly summarized within Appendix). The Autistic SPACE framework, “prioritizes autistic needs and is built on a foundation of domains where the SPACE principles apply (including physical space, processing space, and emotional space).” Dr. Doherty noted her team’s aim is to ensure that the framework is adaptable, where users can freely use it and adapt it to local care settings, such as, for example, emergency departments, geriatric care, or residential/long-term care environments.

### Presentation 3: Autism BrainNet and the Brain Tissue Bank

Dr. David G. Amaral, Scientific Director, Autism BrainNet; Research Director, The MIND Institute; Distinguished Professor, Department of Psychiatry and Behavioral Sciences, University of California, Davis, United States.

Dr. David Amaral presented on the efforts of Autism BrainNet, a program of the Simons Foundation Autism Research Initiative that facilitates and supports scientific research investigating genetic and biological differences of autism and their impact on health, through the study of brain tissue. Highlighting that brain tissue can offer insight into genetic markers and unobservable changes in other tissue, Dr. Amaral stated that continued brain research serves to increase knowledge in the development of improved strategies to support health and quality of life for autistic individuals.

Working with autism organizations, individual and family advocacy groups as well as researchers, Autism BrainNet is a collaborative network of scientific institutions established in 2014 to facilitate the donation process, preservation, and distribution of brain tissue to investigators worldwide on approved scientific studies. While a single donation can support over 100 studies, donations are rare. At the time of the Think Tank, the Autism BrainNet had approximately 148 donations from autistic individuals, but only 12 donations from individuals who had reached the age of 60 years. Dr. Amaral further noted that Autism BrainNet provides support to families throughout the donation process. An initiative, introduced in 2024, allows autistic adults to preregister for brain donation. This process will inform the individual’s family/caregiver(s) of their wish and intention to be a postmortem brain donor.

### Presentation 4: knowledge translation for action

Dr. Fakhri Shafai, Chief Science Officer, AIDE Canada, Canada.

Dr. Shafai presented strategies to optimize knowledge translation to action in our aim of capacity building. She discussed best practices and key considerations when using knowledge and/or research to inspire action and change. Knowing key messages and methods for knowledge mobilization was conveyed as a starting point for creating knowledge translation products that capture attention. Once the message is decided upon, initial steps can be directed to defining the ideal audience and desired outcomes from sharing the information. Determining how to share a message requires the consideration of many factors including time availability and cost, and how to engage the attention of the intended audience.

Dr. Shafai provided guidance on different types of knowledge translation products and the range of time and resource investment required for each. Selected examples from the most-subscribed resources on the AIDE Canada website (www.aidecanada.ca) were provided to illustrate different approaches to sharing a message with a targeted audience (e.g., neurodivergent individuals and their families).

Dr. Shafai discussed best practices for providing a ‘hook’ to an audience, and the importance of visuals and using accessible language. Finally, Dr. Shafai reflected on considerations in conveying the ‘why’ of a message, or the reason a person should care and want to take action based on what they have learned. She posited that appealing to one or more of the following ‘whys’ were most likely to engage the audience: highlighting potential time/cost savings, appealing to a person’s interest, or making an emotional connection. Dr. Shafai suggested that delegates consider these principles when making decisions regarding knowledge translation priorities later in the Think Tank (see ‘Priority Setting Activity’ below).

## Panels

Panels were convened with stakeholders addressing key topics and considerations in aging relative to autism and/or intellectual disabilities. Specific panels addressed: (1) lived experience/self-advocacy, (2) family caregiving/support, (3) quality of life in community, (4) quality of life and health research, (5) specialized healthcare, (6) community health, and (7) primary care. Each panel is summarized below.

### Panel 1: lived experience/advocacy

Panelists: Joanne Gauthier, Sylvère Moulanier, Maxine Share, Terri Robson, and an anonymous panelist: Advocates.

Panelists shared experiences and perspectives of aging relative to autism and/or intellectual disabilities. Health and healthcare were common themes raised, with panelists conveying experiences of frustration, uncertainty, stress, and/or fear in navigating healthcare—unique or heightened challenges for autistic individuals and individuals with intellectual disabilities. They noted changes and complications with aging, and several reflected on the continuing impact of past experiences of trauma on current physical and mental health. Difficulty was described in seeking medical professionals, accessing needed resources, coordinating care, distinguishing symptoms, and processing provided medical information.

Panelists characterized healthcare experiences as largely difficult due to different ways of communicating and interacting. Challenges were noted to include power differentials and communication issues, such as difficulty or a lack of time to ask questions of a doctor or seek more information or other options regarding diagnosis or treatment. Having an advocate in the healthcare encounter was described as critical in order to help ascertain what is being conveyed and pursue additional information as needed.

The necessity for healthcare providers to offer sufficient time with autistic individuals and/or individuals with intellectual disabilities was underscored, along with panel recommendations for more training to healthcare providers about autism and developmental disabilities to better recognize neurodiversity. The panel suggested that better understanding about autism and/or intellectual disabilities among healthcare providers would support greater attentiveness to care needs and improve ways of engagement (e.g., creating space for more communication processing time). Priority areas for development included more knowledge about the potential co-occurrence of physical and/or mental health issues, the development of tools and procedures to support sensory and communication needs, and greater respect for an individual’s agency over their own health and well-being. While recognizing current structural barriers, a panelist recommended that one helpful immediate response of medical professionals could be to simply be open and transparent about lacking autism and/or intellectual disabilities expertise; thus, asking for and incorporating guidance in their practice and in connecting individuals to needed resources.

Quality of life was a common theme highlighted by panelists. Several panelists shared increasing concern about loneliness or isolation among older adults, and emphasized the importance of community connection and meaningful relationships. Capacity to engage in coping strategies (e.g., masking) was identified as a means to avoid difficult social demands and interactions. Panelists also referenced avoiding social connection to decrease perceived “burden” on others. Such reflections may illustrate negative messaging imposed on individuals and internalized stigma, which in turn call for attention to redress ableist orientations in society.

In support of quality of life, panelists addressed the importance of sufficient disability funding and budgeting support to mitigate the risk of poverty. They emphasized that funding support levels should not only provide for basic survival, but also allow for greater quality of life. This was defined as an income level that at least enables individuals to enjoy occasional activities like going to a movie or dining out with friends, without the burden of worrying about covering basic expenses like housing and groceries. For both health and quality of life, the importance of self-advocacy and personal agency was conveyed by the panel. A panelist reminded self-advocates to “never give up!” This conversation further invites exploration of the different meanings of quality of life across autism and/or intellectual disabilities communities, including the consideration of individual, family and cultural perspectives.

### Panel 2: family caregiving/support

Panelists: Jule Hopkins, Wenn Lawson, Tim Stainton, and Lori Watters.

1. Jule Hopkins: Family Advocate, Planned Lifetime Advocacy Network
2. Wenn Lawson, PhD, AFBPsS, MAPs: Autistic Consultant; Independent Researcher; and Adjunct Associate Professor, Curtin University, Western Australia
3. Tim Stainton, PhD: Professor Emeritus, School of Social Work, University of British Columbia; Emeritus Co-Director, Canadian Institute for Inclusion and Citizenship
4. Lori Watters: Advocate

Bringing personal and/or professional experiences supporting neurodivergent individuals, panelists offered considerations related to navigating health/medical, legal and emotional components of aging. They highlighted that the aging process affects all, including families and caregivers, and future planning is an essential component that requires substantial time and intentional effort. Panelists identified the importance of supporting individuals and their caregivers/families through what often is a difficult process of planning for older age, with needed consideration of how to balance the needs of the aging neurodivergent individual, the aging caregiver, and in some cases, a caregiver who also is neurodivergent.

The panel stressed that as adults move into older age, support must be collaborative, with shared responsibility between the individual, their caregiver/family, service/healthcare providers and government/government agency personnel. To this end, it was suggested that tools or resources to support communication with healthcare providers for individuals and their caregivers, would be of use. While the legal and formal pieces of decision-making and addressing basic needs are critically important, panelists also emphasized the importance of advocates and service providers' support in building relationships and facilitating support networks. Respect for individuality and the rallying call of “nothing about us without us” emerged as a key element in building spaces, community connections and services that facilitate quality of life with and for neurodivergent individuals, and in so doing, contribute to peace of mind for caregivers.

### Panel 3: quality of life in community

Panelists: Marie-Joëlle Beaudoin, Nancy Jokinen, Rebecca Pauls, Linda Perry, and Morine Rossi.

1. Marie-Joëlle Beaudoin, PhD(c): Psychology, Professional and Scientific Program, Université du Québec à Montréal
2. Nancy Jokinen, MSW, PhD: Adjunct Professor, School of Social Work, University of Northern British Columbia; Board Director, National Task Group on Intellectual Disabilities and Dementia Practices (NTG); and Co-Lead, NTG-Canada
3. Rebecca Pauls: Executive Director, Planned Lifetime Advocacy Network (PLAN)
4. Linda Perry: Special Projects Coordinator, Vela Canada
5. Morine Rossi: Programs Manager, Autism Edmonton

Panelists conveyed the roles of community service providers and organizations to support aging adults on the autism spectrum and/or with intellectual disabilities. They highlighted a multi-dimensional understanding of the complexities of the aging process for individuals with disabilities and their support networks. Dr. Jokinen noted the importance of establishing a personal record of abilities so that as people age, there is a comparison for them to discuss with their healthcare professional should they begin to experience unexplained changes. As an example, the *NTG-Early Detection Screen for Dementia* tool (https://www.the-ntg.org/ntg-edsd) is being incorporated in annual wellness appointments in the United States.

Support initiatives need to be implemented at population, community and individual levels. Panelists emphasized the importance of fostering opportunities to strengthen relationships and community connections, viewing these commitments as the basis for responsive and individualized supports. Underscoring this discussion was an understanding of the critical role of the community support provider in asking guiding questions and deeply listening for what individuals need rather than expecting them to initiate the identification of their care needs. They suggested that such person- and family-centered support will assist individuals and their caregivers where they’re at. Summarizing, a panelist noted that, “building networks is not only hard work; it is also heart work.”

### Panel 4: quality of life and health research

Panelists: B. Blair Braden, Laura St. John, Gregory L. Wallace, and Agnes H. Whitaker.

1. B. Blair Braden, PhD: Associate Professor, College of Health Solutions, Arizona State University; and Director, Autism and Brain Aging Laboratory
2. Laura St. John, PhD: Assistant Professor, Faculty of Kinesiology, University of Calgary
3. Gregory L. Wallace, PhD: Associate Professor, Department of Speech, Language & Hearing Sciences, The George Washington University
4. Agnes H. Whitaker, MD: Clinical Professor of Psychiatry, Columbia University Irving Medical Center

Building from a shared understanding of how much is still unknown about the impact of aging in autism and/or intellectual disabilities, panelists shared insights from research and clinical practice on means to better understand and support well-being. They reviewed research that highlights the interconnectivity of physical health, mental health, and other issues/considerations. A key recommendation emerging from this session was the need for multi-center, interdisciplinary and international collaboration in longitudinal research so that multi-site data can be collected, shared and mutually used.

The panel further identified concern regarding the current lack of supportive policy addressing the needs of this population, resulting in heightened risk for poor outcomes. While recognizing the necessity of new research, panelists also reflected on what can be done *now* to support this aging population, emphasizing an urgent need for the development and delivery of evidence-based supports that are responsive to individual and community needs and priorities.

### Panel 5: specialized healthcare

Panelists: David G. Amaral, Margaret L. Bauman, Mojdeh Mostafavi, and Gary Stobbe.

1. David G. Amaral, PhD: Professor, Department of Psychiatry & Behavioral Sciences, University of California, Davis; Research Director, The MIND Institute
2. Margaret L. Bauman, MD: Associate Professor, Boston University School of Medicine; Founder, The Autism Research Foundation (TARF), The Autism Research Consortium (TARC), The Lurie Center for Autism (formerly LADDERS), and The Autism Treatment Network (ATN)
3. Mojdeh Mostafavi, MD, ABIM Certified in Internal Medicine: Pediatric Gastroenterology & Nutrition Fellow, Massachusetts General Hospital
4. Gary Stobbe, MD, ABPP: Clinical Professor, University of Washington; Director, Adult Services at Seattle Children’s Autism Center; Medical Director, UW Medicine Adult Autism Clinic; and Director, Adults and Elders Programs, University Center for Excellence in Developmental Disabilities

Focused on specialized healthcare, panelists shared their experiences of providing and/or supporting healthcare to autistic individuals in specific healthcare subspecialty areas. Challenges identified in healthcare delivery were reported to include gaps in healthcare provider knowledge about potential atypical symptom presentation within neurodivergent populations (e.g., communication and cognitive differences, sensory issues, pain threshold, gastrointestinal issues, sleep-related challenges). The panel shared concern about the lack of specialized or general training for healthcare professionals to identify when and how to adapt standardized procedures for neurodivergent individuals. Beyond the need for strategies to integrate training about autism and intellectual disabilities (and other neurodiversities) in medical school and post-graduate training curricula, panelists highlighted the importance of collaboration and building relationships in practice. The effectiveness of interdisciplinary teams was identified as integral to better supporting individuals in navigating the healthcare system, and was seen as key to collaboration in complex care situations. Panelists emphasized the need for policy and remuneration/pay structures that recognize the increased time and human resources needed to assess and provide person-centered care for autistic individuals and/or individuals with intellectual disabilities.

### Panel 6: community health

Panelists: Jennifer Baumbusch, Laura Lee Peters, Brianne Samson, and Jerry Stanger.

1. Jennifer Baumbusch, RN, PhD, FAAN, FCAN: Professor and CIHR Chair in Sex and Gender Science: Dynamics of Caregiving in an Aging Society, University of British Columbia;
2. Laura Lee Peters: Community Resource Advocate, Autism Edmonton
3. Brianne Samson, MOT, Reg. OT (BC): Manager of Services and Community Development-Health, Community Living BC
4. Jerry Stanger, BSW, MHA: Director, Strategic Operations, Community Living BC

Panelists noted that a key component of community health is practicing with intentionality to respect autonomy, self-determination and quality of life—fundamentally recognizing that people have a right to healthcare and community integration. The following aspects were viewed to influence the mental and physical health of autistic adults and/or adults with intellectual disabilities living in the community: (i) preventive healthcare, (ii) suitable housing options including independent living supports, as needed, (iii) access to needed community resources and activity, and (iv) recognition of the crucial role of family caregivers, and attention to needed support for these caregivers.

Accessible and effective health and community services were noted to require sufficient funding, resources and coordination. As stated by a panelist, the issue at hand is not necessarily failure to acknowledge existing needs, but rather the question of who is responsible to comprehensively address what is needed. Panelists identified multiple challenges in the current system, including decreased funding per person, fragmented resources and a lack of individuals trained to address the needs of neurodivergent individuals. Along with structural and policy recommendations to redress barriers to coordinated services, panelists highlighted the importance of innovation and creativity in practice in the aim of quality of life and healthy aging.

### Panel 7: primary care

Panelists: Margaret L. Bauman, David B. Nicholas, Evdokia Anagnostou, and Sandy Stemp.

1. Margaret L. Bauman, MD: Associate Professor, Boston University School of Medicine; Founder, The Autism Research Foundation (TARF), The Autism Research Consortium (TARC), The Lurie Center for Autism (formerly LADDERS), and The Autism Treatment Network (ATN)
2. David B. Nicholas, PhD, RSW: Professor and Associate Dean, Research and Partnerships, Faculty of Social Work, University of Calgary
3. Evdokia Anagnostou, MD: Vice President, Holland Bloorview Rehabilitation Institute; Professor, Faculty of Medicine, University of Toronto
4. Sandy Stemp, BScN: Chief Operating Officer, Reena

Panelists outlined current challenges and recommendations to improve the delivery of healthcare at micro, mezzo and macro levels of the system. Concern was raised that healthcare has primarily focused on younger individuals, with a lack of commensurate attention and resources addressing the needs of older adults in the autism and/or intellectual disabilities communities. Dr. Bauman stated, “it's one of the stresses that those of us who take care of people go through every day. Children are growing up, and I now have folks in my practice who are in their 50’s and 60’s. As a child neurologist, I need to transfer these patients to somebody else, but there isn't anybody else.” It was noted that there is an urgent need for training related to health/mental health and aging for students, residents, and interns to equip them to effectively care for older adults in autism and/or intellectual disabilities communities. Identifying current care challenges, a panelist reflected on geriatrics as a medical specialty and asked the important question, ‘how do we encourage or incentivize medical students to take up that specialty with a focus on neurodivergent individuals – especially when a physician’s time is limited and care for older adults is generally more time consuming and complex than for younger-aged people?’.

It was noted that some individuals in autism and/or intellectual disabilities communities may present with symptoms and localize pain and discomfort differently than non-neurodivergent peers. Non-speaking or minimally verbal individuals were described as being at heightened risk for missed screening and being under-served. Advancing standards for screening and increased training and professional development in this field were deemed to be urgently needed.

Preliminary Canadian data (Nicholas & Anagnostou, in development) appear to indicate that some older autistic adults experience challenges with executive function and healthcare navigation, including making appointments and accessing care. Multiple individuals in this study report negative healthcare experiences, including sensory issues and trauma, but they also demonstrate resilience. In identifying core healthcare issues that need to be addressed, a pressing question for researchers and practitioners was, ‘what should we be measuring to assess baseline functioning and shifts in physical health, mental health and resource needs?’ Substantial investment in research on aging in autism and/or intellectual disabilities was strongly recommended.

Panelists reflected on the intersection of health and developmental services in advancing needed resources for this population. In Ontario, Canada, as an example, a partnership was developed over many years between the intellectual/developmental disabilities, aging, and long-term care sectors. Such partnership was conveyed as important as a means to avoid duplication of services and to advance collective action, thus inviting a community development approach built on cross-sectoral engagement. Representation from various levels of government was recommended, along with academics/researchers and representatives in areas such as health, seniors/aging, social services, housing, etc. Questions emerged related to supportive aging in place; for instance, ‘how do we integrate health, healthcare and proactive housing models?’ In considering these various important questions, the panel stressed that access to needed healthcare is a human right; hence, proactive action is urgently required.

## Priority setting activity

Following information sharing and substantial deliberation among Think Tank delegates that included: (1) a review of Canadian primary care guidelines for adults with intellectual and developmental disabilities [17] and the “Autistic SPACE” framework items [19], (2) an overview of aging in autism and intellectual disabilities scoping review findings, and (3) keynote addresses, presentations and panel discussions (as summarized above), delegates were invited to reflect on and rank priorities for near-term (within two years) resource development and knowledge translation, with the ultimate aim of ameliorating healthcare and community services for older adults on the autism spectrum and/or with intellectual disabilities. Using a vote-counting technique, delegates could identify up to 6 guidelines/framework items that were perceived to most warrant near-term resource development/knowledge translation. Delegates thus had the opportunity to select individual guidelines and framework items presented by Sullivan et al. [17] or Doherty et al. [19], respectively. Voting on priorities reflected each delegate’s appraisal of the most pressing needs for resource development/knowledge translation over the near-term. Total votes thus were indicative of the most commonly-identified priorities. Table 1 outlines the ranking results, with further explanation below.

**Table 1:** Ranking of priority for near-term knowledge translation/capacity building

(1) Knowledge Translation Priority D: Doherty et al. [19] framework item; S: Sullivan et al. [17] guideline (Note: items & guidelines adapted and/or truncated)	(2) All Groups	(3) Self Advocates	(4) Caregivers/Supporters	(5) Community Service Providers	(6) Clinical/ Healthcare Providers
D1 Attention to sensory needs	11	7	0	2	2
D2 Predictability	6	3	0	2	1
D3 Acceptance	5	3	0	1	1
D4 Communication S2 Effective communication	22	14	2	3	3
D5 Empathy	9	4	3	2	0
D6 Ensure sufficient (“wider”) space in care	8	5	3	0	0
S1 Person-centered care approach	37	15	11	6	5
S3 Capacity for decision making (to extent possible)	14	6	8	0	0
S4 Caregivers/families	17	0	15	2	0
S5 Interprofessional healthcare teams to support the range of health and developmental needs	34	0	8	16	10
S6 Health assessments and preventive care	35	12	5	8	10
S8 Cognitive ability and adaptive functioning	6	0	0	0	6
S9 Pain and distress: May manifest differently	13	1	4	1	7
S10 Polypharmacy and long-term use of certain medications	11	0	1	10	0
S11 Abuse, exploitation and neglect (emotional, verbal, physical, sexual, financial)	17	4	2	10	1
S12 Life transitions (e.g., transition to/in adulthood, frailty, end-of-life)	19	3	10	3	3
S26 & 27 Mental health	14	1	3	4	6
S28 & 29 Psychiatric disorders	3	0	0	0	3
S31 Addictions	0	0	0	0	0
S32 Dementia	17	1	1	6	9

Each of the reviewed framework items [19] and care guidelines [17] are itemized in the first column of Table 1 (with brief definitions in Appendix). Column 2 provides the total number of votes per item/guideline which indicates overall ranking of each item/guideline’s level of priority or urgency for near-term resource development/knowledge translation. The most frequently prioritized framework items/guidelines warranting resource development/knowledge translation were: a person-centered approach (*n* = 37), health assessments and preventive care (*n* = 35), interprofessional healthcare teams to support the range of health and developmental needs (*n* = 34), communication (*n* = 22), and life transitions (e.g., to/in adulthood, frailty and end-of-life) (*n* = 19), families/caregivers (*n* = 17), abuse, exploitation and neglect (emotional, verbal, physical, sexual, financial) (*n* = 17), dementia (*n* = 17), and mental health/psychiatric disorders (combined categories) (*n* = 17). It is important to note that some indicated items/guidelines overlap (e.g., person-centered care likely includes other items such as communication and empathy); hence, caution must be exercised in drawing definitive conclusions from this vote counting. Rather, this approach was used to stimulate and refine further conversation and exploration relative to resource development priorities. Also, it was noted that framework items/care guidelines that were less subscribed, i.e., less voted for, do not necessarily imply that they were viewed to be unimportant or less important in terms of the need for resource development. Rather, the higher rankings of items/guidelines indicated their prioritization for *near-term* resource development.

Delegates were further invited to specify the target audience or group to which near-term resource development/knowledge translation (specific to a given framework item or care guideline) would most benefit and/or be best targeted to, i.e., self-advocates, family caregivers/supporters, community services providers, and clinical/healthcare providers. Delegates were asked to indicate one group that was viewed to most benefit from a given resource. However, it was acknowledged that in some cases, multiple groups likely would benefit from a given knowledge translation/capacity building resource. Results from this latter inquiry are summarized in Columns 3 to 6 in Table 1.

For individuals with lived and living experience, termed ‘self-advocates’, the items prioritized for near-term resource development/knowledge translation were: person-centered care (*n* = 15), communication in care (*n* = 14), and health assessments and preventive care (*n* = 12). For family caregivers/supporters, areas most frequently identified for near-term resource development/knowledge translation comprised: support to caregivers and families (*n* = 15), a family-centered care approach (*n* = 11), and life transitions (e.g., to/in adulthood, frailty and end-of-life, *n* = 10). For community service providers, areas most frequently indicated for near-term resource development/knowledge translation were: interprofessional healthcare teams to support the range of health and developmental needs (*n* = 16), polypharmacy and long-term use of certain medications (*n* = 10), and addressing abuse, exploitation and neglect (emotional, verbal, physical, sexual, financial) (*n* = 10). For clinical/healthcare providers, the most frequently indicated areas for near-term resource development/knowledge translation were: interprofessional healthcare teams to support the range of health and developmental needs (*n* = 10), health assessments and preventive care (*n* = 10), and dementia (*n* = 9).

Specific diagnostic areas or conditions identified by Sullivan and colleagues [17; item #13 in Appendix] were reviewed, with the aim of identifying aging-related clinical conditions about which near-term resource development/knowledge translation should be prioritized to support care to autistic individuals and/or individuals with intellectual disabilities. Again, based on vote counting, delegates indicated the highest priorities for near-term resource development for healthcare providers to be the following physical healthcare areas/specialties (Note: mental health was not included in voting options): (i) arthritis and/or bone health, (ii) cancer screening and treatment, (iii) cardiometabolic care, (iv) emergency care, (v) gastrointestinal functioning, (vi) gender and LGBTQIA + health, (vii) neurology, (viii) pain management, (ix) palliative care, (x) reproductive health, and (xi) sleep challenges. Some priority areas were not specifically indicated in the primary care guidelines [17], but were added or adapted by Think Tank delegates in a consensus-building process. Resource development and capacity building were noted to be critical for improving care for older autistic adults and/or adults with intellectual disabilities.

## Discussion

Advancing partnership and developing resources for practice advancement emerged as important priorities. Attention to a range of issues across individual, caregiver, health, community and policy levels, was emphasized. Delegates reflected on considerations such as experiences of aging and healthcare in autism and intellectual disabilities; risks for physical and mental health issues; environmental, sensory and/or communication considerations; the need for supports; the imperative of individual agency and human rights; quality of life; and the urgency of care provider and system capacity building.

Critically reflecting on whose perspectives are being heard (versus are not being heard) was highlighted, with a call for inclusion and representation across the diversities in the autism and intellectual disabilities communities. Trauma-informed care and building infrastructures of policy and programming for individuals and their caregivers/supporters emerged as urgently needed. Such advancement was deemed critical in community and health services, with a further call for intentionality in addressing intersectionality and social determinants of health barriers.

In moving forward, there was a strong commitment to recognize and support (and not duplicate) the important work of various groups nationally and internationally that are advancing support, resources and systems for aging neurodivergent individuals. As an example, the National Task Group (NTG) on Intellectual Disabilities and Dementia Practices guidelines for community care and support for people with intellectual disabilities affected by dementia [21] recently updated guidance and resources [22, 23]. This initiative offers important guidance related to community members affected by dementia and illustrates information that needs to be integrated in care.

Collaboration between autism-specific organizations and mainstream aging services is recommended to better support older autistic and/or intellectually disabled adults. This invites linkages with communities, governments, clinicians, and other partners in fields such as health/mental health, community living, housing, long-term care, advanced education, employment supports, transition planning, urban/municipal planning and justice. Within such efforts, adhering to a human rights framework is paramount in upholding the right to age with agency and support commensurate to needs and preferences. Such a commitment requires advocacy and capacity building, including policy development, training, practice standards, funding and mentoring.

Delegates identified collective commitment to collaboration for advancing knowledge in neurodivergence and later life. Practical suggestions emerging from the Think Tank comprised ongoing engagement for resource development and knowledge translation products, and extending our partnership.

## Calls to action

The following *Calls to Action* were identified to address the needs of older autistic adults and individuals with intellectual disabilities via capacity building. The symbiotic nature of concurrently using top-down and bottom-up strategies and the development of guiding strategies were recognized as important to move to proactive action.

1. *Recognize autism and intellectual disabilities as continuing aspects of the human lifespan*. Aging is an integral part of the human condition. Yet there is limited understanding of how aging impacts neurodivergent communities such as autistic individuals and/or individuals with intellectual disabilities. Societal investment is needed in aging-based research, services and supports to identify and meet the priorities and needs of aging neurodivergent individuals.
2. *Prioritize the foundational principle of ‘nothing about us without us.’* Neurodivergent perspectives must be key contributors to ground initiatives in research, clinical and community care and policy. Attention to diversities such as sex, gender, ethnicity, region, co-existing conditions and other considerations is essential for greater intersectional representation. Accessibility is a key component to realize meaningful representation and participation.
3. *Increase the volume, depth and quality of research*. Greater understanding is warranted about how autism and intellectual disabilities intersect with quality of life and mental and physical health in aging. Developing research standards and processes for comparative datasets is anticipated to expedite knowledge generation. International research could be developed through participatory design as well as consideration of means to exponentially increase knowledge advancement such as data/information exchange to maximize data utility. This could be orchestrated by a clearinghouse or center that gathers and supports this research development and sharing.
4. *Invest in aging with dignity, agency and community*. Navigating physical, mental health, legal, financial, and social changes that accompany life transitions can present challenges for older neurodivergent adults and their supporters. Investment is thus needed for developing resources that can better support adults and enhance services and system navigation. Access to sufficient healthcare and community supports, financial security and means to ease loneliness, emerged as key self-advocate priorities. Societal investment in improved care, housing, services and socialization opportunities is essential to facilitate aging in community with agency and choice.
5. *Build integrated interprofessional and intersectoral relationships*. Quality of life and health are interconnected, yet resources and service providers are often siloed. Collaboration opens opportunities to improve access to services and supports, learn from other sectors and build partnered innovation. A broader systemic approach to optimize well-being in the autism and/or intellectual disabilities communities is contingent on broader systemic capacity building in relevant sectors (e.g., community planning, first responders, health, mental health, housing, transportation, long-term care, etc.).
6. *Training of health and mental health trainees and practitioners about neurodivergence and aging*. Education is warranted to increase care provider knowledge about the experiences and physical and mental healthcare needs of neurodivergent people, as well as evidence-informed means to address these needs. Presentation of symptoms and care strategies are examples of practice areas to advance.
7. *Develop practices, protocols, tools, and policies to support health assessments and monitoring*. With aging, autistic adults and/or adults with intellectual disabilities may experience complex and overlapping health concerns. Increased evidence-informed resources and approaches are needed to improve screening, diagnosis and treatment of health issues.

## Limitations

The Think Tank offered opportunity for collective reflection on aging in autism and/or intellectual disabilities. While attempts were made to inclusively invite a range of representatives and leaders in this field to the Think Tank, we were restricted by budget and delegate availability. It is important to acknowledge that this meeting included a limited number of perspectives, inviting caution in considering key messages conveyed and in particular, the ranking/prioritizing of items perceived to warrant near-term resource advancement.

Think Tank content was in part orchestrated around selected care guidelines and a framework offered in the literature. While this orientation was helpful in building on previous scholarship and moving toward actionable next steps in resource development (indeed, a key aim of the Think Tank), it is acknowledged that these pre-determined notions, as presented in this proceedings report, may have influenced and perhaps constrained broader thinking. On the other hand, the utilized sources offered rich fodder for reflection and discussion, presented diversity across developmental disabilities [17] and autism [19], and offered perspectives from diverse world regions. It is also acknowledged that the two utilized sources, i.e., care guidelines and framework items, focus broadly on adulthood—not specifically *older* adulthood—which invites further research as well as frameworks and guidelines particular to autism and/or intellectual disabilities in later years.

Our voting exercises informed steps toward determining resource development priorities. However, as noted above, some of the identified framework and guideline items blended conceptual notions which may have impeded discrete or distinct options in delegate voting. Accordingly, the presented priorities need to be considered only as a preliminary step and thus, viewed with caution. To that end, further work, beyond this meeting, is underway in refining priorities for resource development and capacity building.

Finally, we acknowledge an important limitation of integrating autism and intellectual disabilities within our substantive focus. While commonalities and overlap exist within and across autism and intellectual disabilities, so too do significant distinctions that need to be carefully considered. Further work is advised, with attention to granularity per community.

## Conclusion

The Think Tank was generative in bringing colleagues together to reflect on pressing considerations and preliminary priorities for near-term resource development. After deliberating on aging in autism and intellectual disabilities in the Think Tank, there was a strong consensus that much work is needed at multiple levels. Focusing on later life as a critically important yet under-addressed ‘age and stage’ in autism and intellectual disabilities, invites evidence generation and proactive action and change.

## Data Availability

Not applicable.

## Appendix

**Table 2:** Care guidelines/framework items reviewed at the Think Tank; developed by Doherty et al. [19] and Sullivan et al. [17], and integrated and adapted in this table

**ISSUE/NEED ADDRESSED**	**GUIDELINE/FRAMEWORK DEFINITION (Adapted)**
**Doherty et al: ** **1 - Sensory Needs:** Environmental sources of stress noted to be “[i]mperceptible to non-autistic people” (p. 2), hence distress may not be discerned.	**•** Need to better understand and attend to sensory differences; sight, sound, smell, taste, touch, temperature, proprioception, introception and pain (differences interpreting internal bodily functions [e.g., pain, hunger, thirst, weariness, needing to use the restroom])
**Doherty et al: 2 -Predictability **	**•** Maximize predictability (accurate information shared in advance) **•** Ensure information/resources for optimal physical environment (e.g., signage), clarity of consultation (e.g., use of photos/videos), planning relative to procedures, waiting in conducive/familiar environment (e.g., one’s vehicle), familiar provider
**Doherty et al: 3 - Acceptance**	**•** Acknowledgement that making “Autistic people appear non-autistic can be deeply harmful” (p. 3) **•** Understand/accommodate/accept various ways of engaging in health information **•** Training in/understanding behaviors/responses (e.g., stimming, monotropic thinking patterns) **•** Provide detailed information, as needed **•** Understand signs of distress
**Doherty et al: 4 - Communication** **Sullivan et al: 2 - Effective communication**	**•** Attend to varying communication support needs (e.g., Augmentative and Alternative Communication) **•** Clear and direct communication **•** Understand and accept/accommodate potential communication differences (e.g., eye contact, gestures, posture, differences in indicating distress or pain, potential temporary inability to communicate verbally, if overwhelmed)
**Doherty et al: 5 - Empathy**	**•** Need to better understand the individual’s perspectives **•** Ask the individual about their understanding of issues **•** Check verbal comprehension **•** Ensure shared understanding of treatment plans/goals
**Doherty et al: 6 - Ensure Sufficient (“Wider”) Space in Care**	**•** Physical Space: Respect the individual’s comfort with “touching distance” (p. 5); attend to tactile defensiveness; avoid “noxious stimuli” from the perspective of the individual (p. 5) **•** Processing Space: Ensure sufficient time to process information or changes: “It may appear that an autistic person is not answering or did not understand, and so the temptation is to repeat or rephrase the question, both of which can re-start the processing time thus further delaying resolution [24]” (p. 5). **•** Emotional Space: Understand and accommodate the challenge of identifying, processing and managing emotions, including impacts of sensory overload or emotional overwhelm. If the individual is overwhelmed, “ensure safety, minimize environmental sensory input, and offer no unnecessary words – providing space and time to recover” (p. 5).
**Sullivan et al: 1 - Person-centered care approach**	**•** May require additional time and supports to address individual needs **•** With the individual, identify someone who knows them well (accompanies, coordinates care and/or monitors ongoing needs) **•** Provide sufficient time and support to ensure needs and perspectives are understood **•** Collaborate with the individual and their caregiver regarding goals and values of care
**Sullivan et al: 2 - Effective communication**	• Directly address the individual **•** Engage the individual, including attending to verbal and nonverbal cues **•** Slow down communication **•** Involve caregivers known to the individual to assist with communication, but watch for “inappropriate” influence over decision making **•** Have brief times alone with the individual to attend to safety or concerns
**Sullivan et al: 3 - Capacity for decision making (key issues in participating to the extent possible)**	**• **Assess capacity for decision making, need for accommodations/supports (can use “Decision-Making Checklist”) **•** Assess capacity, adapt communication, as needed, and involve family/caregiver **•** Engage a “shared decision making process”, as needed (p. 259)
**Sullivan et al: 4 - Families and other caregivers**	**•** Regularly screen/assess for support needs of caregivers, and recommend supports as needed
**Sullivan et al: 5 - Interprofessional healthcare teams: Support range of health and developmental needs**	**•** Ensure integrated team of healthcare professionals ideally familiar with adults with intellectual and developmental disabilities **•** Have designated leader/coordinator
**Sullivan et al: 6 - Health assessments and preventive care**	**•** Periodic health assessments using established guidelines (e.g., primary care for people with intellectual and developmental disabilities, adapted tools) **•** Monitor and seek improved outcomes (e.g., engage individuals with intellectual and developmental disabilities, and staff; align assessment and care) **•** Develop a health action plan based on assessment, determine priorities and timelines, share copy of the plan with the individual and their caregiver
**Sullivan et al: 8 - Cognitive ability and adaptive functioning**	**•** For unexplained behavioral shifts in individuals unable to communicate directly, (re)assess cognitive ability and adaptive functioning to determine the root cause of change to determine support needs
**Sullivan et al: 9 - Pain and distress: may manifest differently and if communication differences, more difficult to detect**	**•** Engage caregiver and adapted tools to assess pain and its intensity **•** Integrate comprehensive, systematic approach to assess "behaviors that challenge" relative to indicating pain or distress (p. 261) **•**"Common sources of pain include injury, dental caries, GERD (gastroesophageal reflux disease), arthritis, constipation, and dysuria. Distress can be a response to pain, challenges in the person’s environment (e.g., sensory hypersensitivity), lack of supports, or some negative life experiences"(p. 261).
**Sullivan et al: 10 - Polypharmacy and long-term use of certain medications**	**•** Regularly review (every 3 months) medications (initiation date, indications, dose effectiveness, adverse/unwanted impacts); involve pharmacist, as possible **•** If medications potentially toxic or with drug-organ or drug-drug interactions, determine baseline for patient, with recommended intervals of monitoring **•** Provide education to the individual and their caregiver re. medication; determine individual’s capacity and need for support in decision making and medication adherence **•** Simplify medication use
**Sullivan et al: 11 - Abuse, exploitation and neglect (emotional, verbal, physical, sexual, financial)**	**•** Assess for risk **•** Report suspected abuse, exploitation or neglect to relevant authorities **•** Consider resources with expertise in this population
**Sullivan et al: 12 - Life transitions (transition to adulthood, frailty, end-of-life)**	**•** Proactively address transitions and potential role shifts with the individual, their caregiver, and healthcare team members **•** Offer psychosocial supports **•** Collaborate with the individual, their caregiver and care partners in the development of a “transition plan, comprehensive medical summary,… and transition readiness checklist” (p. 262)
**Sullivan et al: 26 & 27 - Mental health**	**•** Assess “behaviors that challenge” (#27, p. 268) (e.g., self-injury, aggression, anger outbursts, irritability) for underlying cause **•** Consider needed accommodations in one’s environment **•** Consider potential cumulative impacts of co-occurring conditions, adverse life events, and/or environmental perceptions on mental health
**Sullivan et al: 28 & 29 - Psychiatric disorders**	**•** Be aware of increased vulnerability to mental health stresses **•** Screen for “antecedents, life events, and other triggers of mental distress” (p. 268) **•** Assess and plan proactively and offer supportive intervention **•** Consult re. co-occurring issues/conditions **•** Engage interprofessional team and the individual/caregiver to optimize communication **•** Engage mental health team for intervention plan
**Sullivan et al: 31 – Addictions**	**•** Need for targeted assessment and support
**Sullivan et al: 32 – Dementia**	**•** Be aware of diagnostic challenges due to potential gradual and subtle "changes in emotion, social behavior, or motivation" (p. 271) **•** Investigate possible reversible causes **•** Need for “a baseline assessment of cognitive, adaptive, and communicative functioning” (p. 271): Suggested at age 40 yrs., and for people with Down Syndrome, at age 30 yrs.
**Sullivan et al: 13 - Physical Health Areas (including #14-25)**	**•** Physical inactivity and obesity **•** Vision and hearing impairments **•** Oral disease **•** Cardiovascular disease **•** Respiratory disease **•** Gastrointestinal problems **•** Women’s gynecologic and reproductive health **•** Neuromuscular and skeletal disorders **•** Epilepsy **•** Endocrine disorders **•** Infectious disease **•** Cancer screening **•** Sleep problems
